# The genome sequence of the plain-faced dronefly, *Eristalis arbustorum *(Linnaeus, 1758)

**DOI:** 10.12688/wellcomeopenres.17580.1

**Published:** 2022-02-15

**Authors:** William Hawkes, Karl Wotton, University of Oxford and Wytham Woods Genome Acquisition Lab, Darwin Tree of Life Barcoding collective, Wellcome Sanger Institute Tree of Life programme, Wellcome Sanger Institute Scientific Operations: DNA Pipelines collective, Tree of Life Core Informatics collective, Darwin Tree of Life Consortium

**Affiliations:** 1 Department of Biosciences, University of Exeter, Penryn, TR10 9FE, UK

**Keywords:** Eristalis arbustorum, plain-faced dronefly, genome sequence, chromosomal, Diptera

## Abstract

We present a genome assembly from an individual female *Eristalis arbustorum* (the plain-faced dronefly; Arthropoda; Insecta; Diptera; Syriphidae). The genome sequence is 451 megabases in span. The majority of the assembly (94.71%) is scaffolded into 6 chromosomal pseudomolecules, with the X sex chromosome assembled. The complete mitochondrial genome was also assembled and is 16.0 kilobases in length.

## Species taxonomy

Eukaryota; Metazoa; Ecdysozoa; Arthropoda; Hexapoda; Insecta; Pterygota; Neoptera; Endopterygota; Diptera; Brachycera; Muscomorpha; Syrphoidea; Syrphidae; Eristalinae; Eristalini; Eristalis; *Eristalis arbustorum* (Linnaeus, 1758) (NCBI:txid1124515).

## Background

The plain-faced drone fly, *Eristalis arbustorum*, is a smaller member of the *Eristalis* genus. Both sexes lack an obvious median stripe down the centre of their face (which *E. nemorum* has) and have more plumose arista and darker tips of the mid tibiae than *E. abusiva* ( van Veen, 2010). Like others in the *Eristalis* genus, *E. arbustorum* has large pale patches either side of its abdomen and is a batesian mimic of the honeybee Apis mellifera to gain protection from predators such as birds. This mimicry is not limited solely to visual similarities but also behavioural and acoustic similarities ( Golding & Edmunds, 2000; Moore & Hassall, 2016). *E. arbustorum* is widespread and common in the UK, and can be found in a variety of open habitats feeding on a range of flowers including Apiaceae and Asteraceae, making it an important generalist pollinator ( Ball *et al.*, 2015; Doyle *et al.*, 2020). The larvae of *E. arbustorum* are colloquially known as rat-tailed maggots and feed on decaying organic matter in organically rich pools ( Ball *et al.*, 2015; van Veen, 2010). Interestingly, as in *E. tenax*, reproduction by larval stages (paedogenesis) has been suggested to occur ( Achterkamp *et al.*, 2000) and *E. arbustorum*, along with other other *Eristalis* flies, play a highly important ecological role in terms of decomposition ( Hurtado *et al.*, 2008). *E. arbustorum* has also been used as a model for the plastic response to temperature on traits such as colour pattern and wing size and has featured in studies of fine scale population structure ( Francuski *et al.*, 2020; Ottenheim *et al.*, 1998). This is the first production of a high quality *E. arbustorum* genome and we believe that the sequence described here, generated as part of the Darwin Tree of Life project, will further aid understanding of the biology and ecology of this hoverfly.

## Genome sequence report

**Figure 1.**
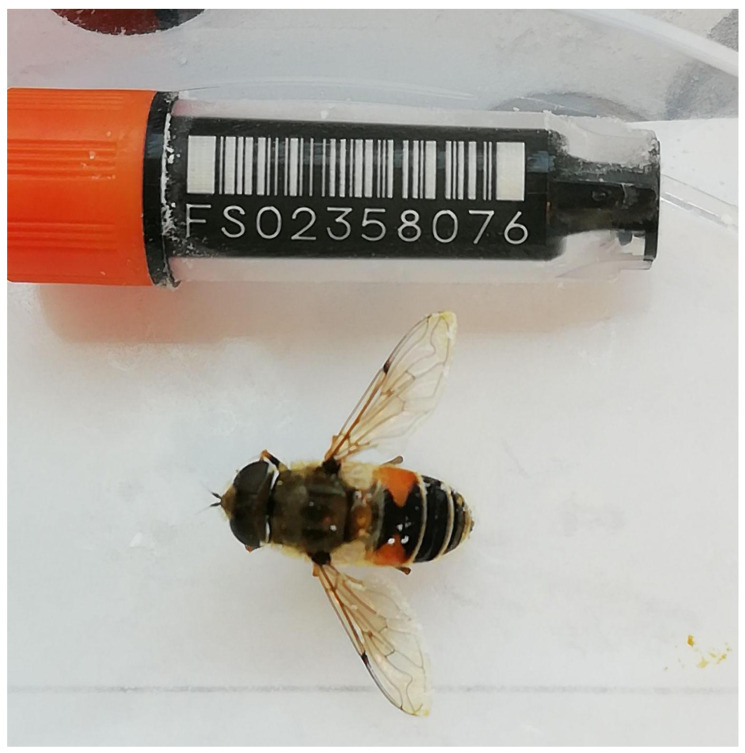
Image of the *Eristalis arbustorum* specimen used in genome sequencing taken during preservation and processing.

The genome was sequenced from a single female *E. arbustorum* collected from Wytham Great Wood, Oxfordshire, UK (latitude 51.769, longitude -1.330) ( Figure 1). A total of 19-fold coverage in Pacific Biosciences single-molecule long reads and 87-fold coverage in 10X Genomics read clouds were generated. Primary assembly contigs were scaffolded with chromosome conformation Hi-C data. Manual assembly curation corrected 370 missing/misjoins and removed 5 haplotypic duplications, reducing the assembly size by 0.11% and scaffold number by 33.59%, and increasing the scaffold N50 by 244.23%.

**Figure 2.**
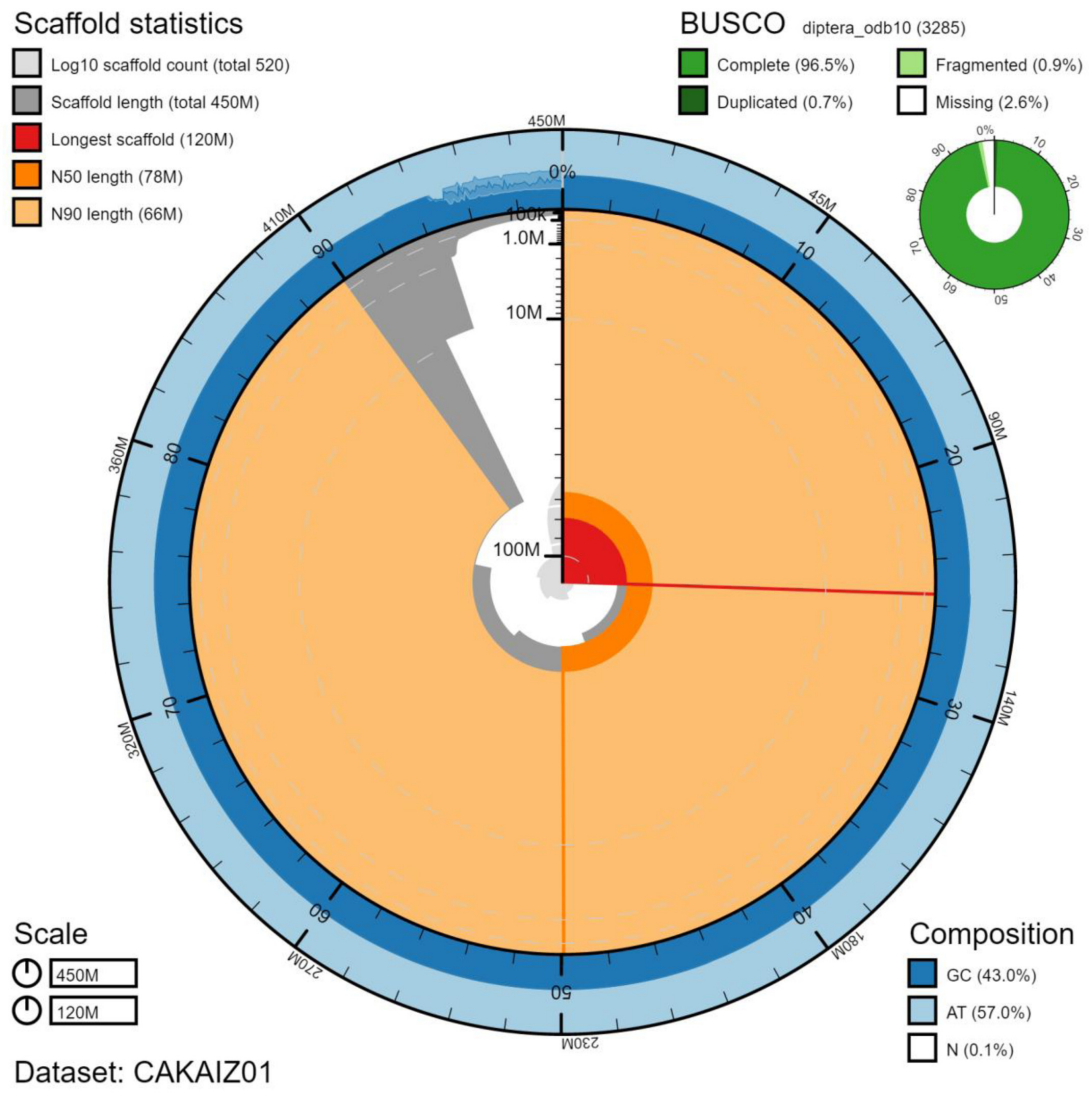
Genome assembly of *Eristalis arbustorum*, idEriArbu1.1: metrics. The BlobToolKit Snailplot shows N50 metrics and BUSCO gene completeness. The main plot is divided into 1,000 size-ordered bins around the circumference with each bin representing 0.1% of the 451,042,988 bp assembly. The distribution of chromosome lengths is shown in dark grey with the plot radius scaled to the longest chromosome present in the assembly (115,465,858 bp, shown in red). Orange and pale-orange arcs show the N50 and N90 chromosome lengths (78,272,982 and 66,425,613 bp), respectively. The pale grey spiral shows the cumulative chromosome count on a log scale with white scale lines showing successive orders of magnitude. The blue and pale-blue area around the outside of the plot shows the distribution of GC, AT and N percentages in the same bins as the inner plot. A summary of complete, fragmented, duplicated and missing BUSCO genes in the diptera_odb10 set is shown in the top right. An interactive version of this figure is available at https://blobtoolkit.genomehubs.org/view/idEriArbu1.1/dataset/CAKAIZ01/snail.

The final assembly has a total length of 451 Mb in 257 sequence scaffolds with a scaffold N50 of 77.5 Mb ( Table 1). The majority, 94.71%, of the assembly sequence was assigned to 6 chromosomal-level scaffolds, representing 5 autosomes (numbered by sequence length), and the X sex chromosome ( Figure 2– Figure 5; Table 2). Based on published karyotype potential micro-chromosomes have not been recovered in the curated assembly ( Rozek *et al.*, 1995) The assembly has a BUSCO v5.1.2 ( Manni *et al.*, 2021) completeness of 96.5% (single 95.9%, duplicated 0.7%) using the diptera_odb10 reference set. While not fully phased, the assembly deposited is of one haplotype. Contigs corresponding to the second haplotype have also been deposited.

**Table 1.** Genome data for *Eristalis arbustorum*, idEriArbu1.1.

*Project accession data*	
Assembly identifier	idEriArbu1.1
Species	*Eristalis arbustorum*
Specimen	idEriArbu1 (PacBio, 10X, Hi-C); idEriArbu2 (RNAseq)
NCBI taxonomy ID	1124515
BioProject	PRJEB46302
BioSample ID	SAMEA7520036
Isolate information	Females, head/thorax (idEriArbu1), abdomen (idEriArbu2)
*Raw data accessions*	
PacificBiosciences SEQUEL II	ERR7057611
10X Genomics Illumina	ERR6787423-ERR6787426
Hi-C Illumina	ERR6787428
Illumina PolyA RNAseq	ERR6787427
*Genome assembly*	
Assembly accession	GCA_916610145.1
*Accession of alternate haplotype*	GCA_916610155.1
Span (Mb)	451
Number of contigs	1,338
Contig N50 length (Mb)	1.2
Number of scaffolds	518
Scaffold N50 length (Mb)	78.3
Longest scaffold (Mb)	115.5
BUSCO * genome score	C:96.5%[S:95.9%,D:0.7%],F:0.9%,M:2.6%,n:3285

*BUSCO scores based on the diptera_odb10 BUSCO set using v5.1.2. C= complete [S= single copy, D=duplicated], F=fragmented, M=missing, n=number of orthologues in comparison. A full set of BUSCO scores is available at https://blobtoolkit.genomehubs.org/view/idEriArbu1.1/dataset/CAKAIZ01/busco.

**Figure 3.**
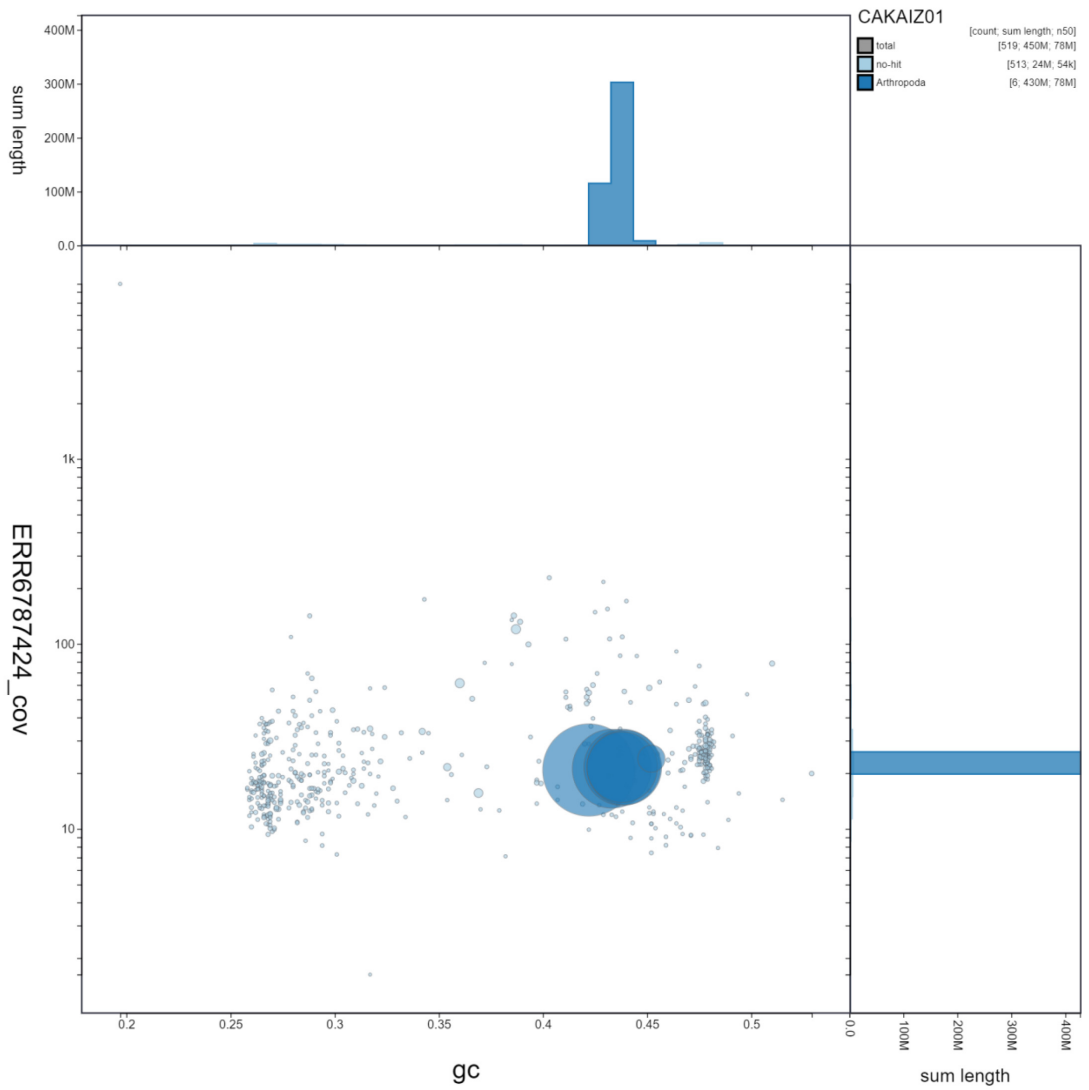
Genome assembly of *Eristalis arbustorum*, idEriArbu1.1: GC coverage. BlobToolKit GC-coverage plot. Scaffolds are coloured by phylum. Circles are sized in proportion to scaffold length. Histograms show the distribution of scaffold length sum along each axis. An interactive version of this figure is available at https://blobtoolkit.genomehubs.org/view/idEriArbu1.1/dataset/CAKAIZ01/blob.

**Figure 4.**
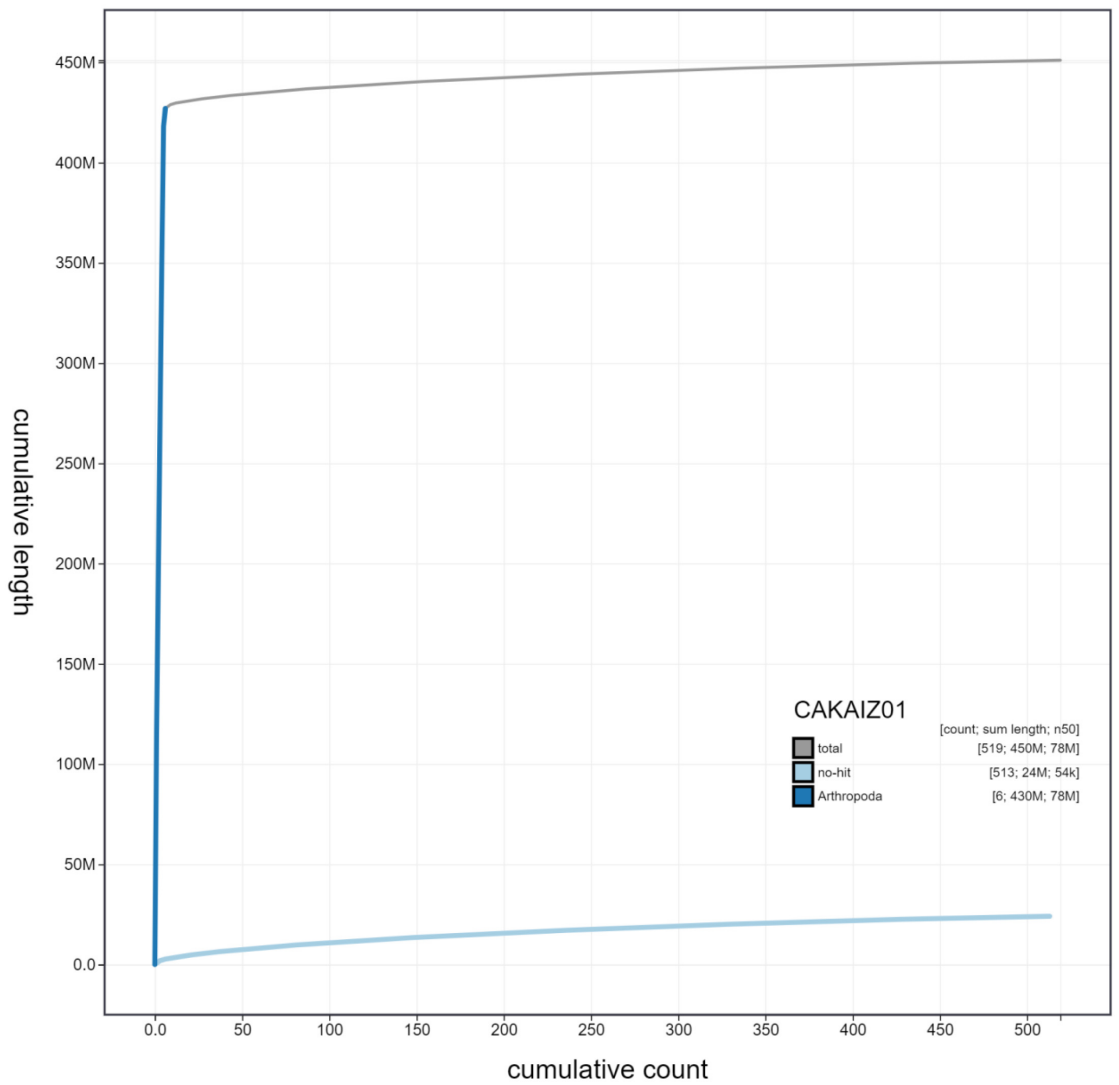
Genome assembly of *Eristalis arbustorum*, idEriArbu1.1: cumulative sequence. BlobToolKit cumulative sequence plot. The grey line shows cumulative length for all scaffolds. Coloured lines show cumulative lengths of scaffolds assigned to each phylum using the buscogenes taxrule. An interactive version of this figure is available at https://blobtoolkit.genomehubs.org/view/idEriArbu1.1/dataset/CAKAIZ01/cumulative.

**Figure 5.**
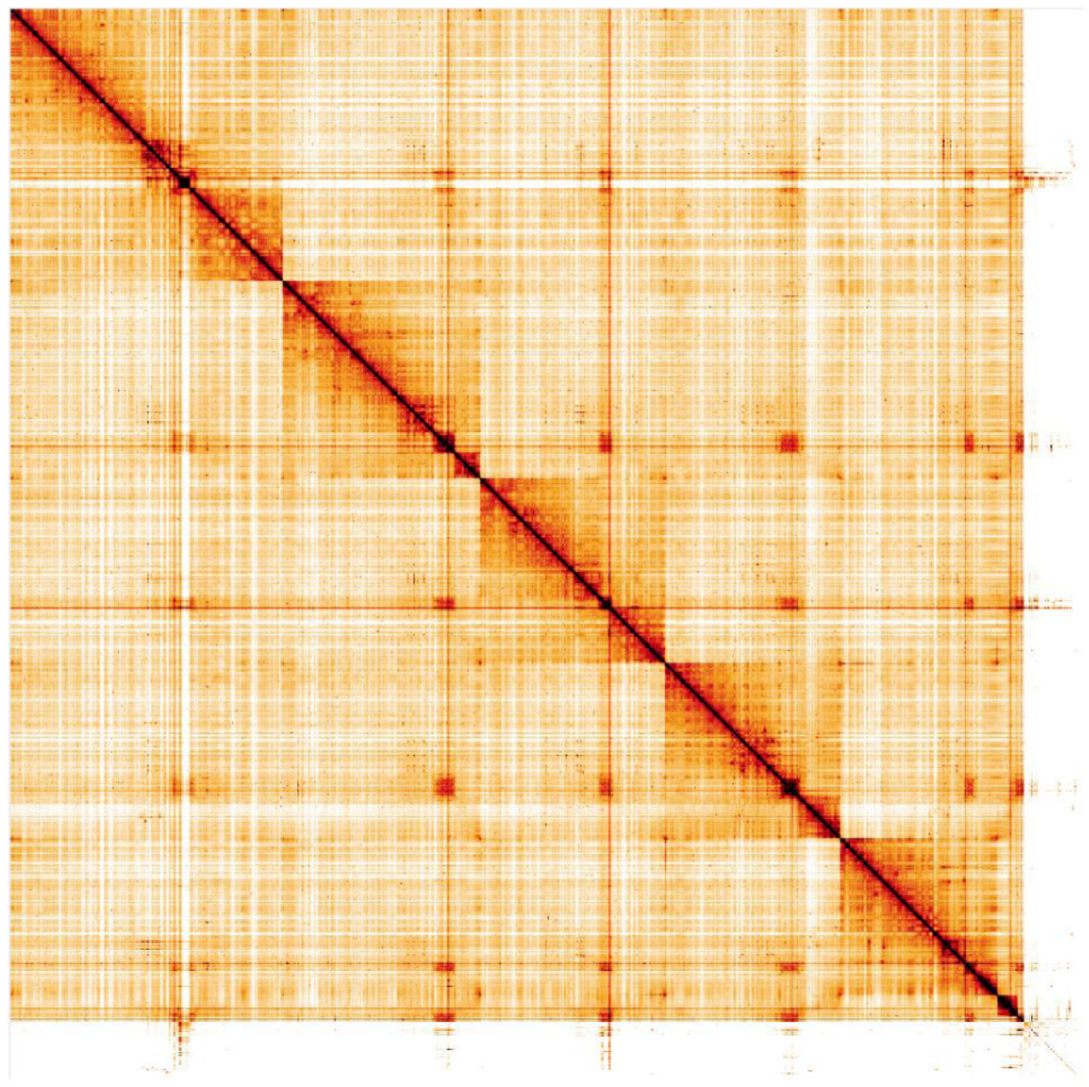
Genome assembly of *Eristalis arbustorum*, idEriArbu1.1: Hi-C contact map. Hi-C contact map of the idEriArbu1.1 assembly, visualised in HiGlass. Chromosomes are arranged in size order from left to right and top to bottom.

**Table 2.** Chromosomal pseudomolecules in the genome assembly of *Eristalis arbustorum*, idEriArbu1.1.

INSDC accession	Chromosome	Size (Mb)	GC%
OU744316.1	1	115.47	42.2
OU744317.1	2	83.54	43.3
OU744318.1	3	78.27	43.8
OU744319.1	4	74.46	43.9
OU744320.1	5	66.43	43.8
OU744321.1	X	8.83	45.2
OU744322.1	MT	0.02	19.7
-	-	24.04	37.0

## Methods

### Sample acquisition and nucleic acid extraction

Two female *E. arbustorum* samples, idEriArbu1 and idEriArbu2, were collected from Wytham Great Wood, Oxfordshire, UK (latitude 51.772, longitude -1.339) by Will Hawkes, University of Exeter on 7 (idEriArbu1) and 8 (idEriArbu2) August 2019. The specimens were caught with a net, snap-frozen on dry ice and stored using a CoolRack.

DNA was extracted from the head/thorax of idEriArbu1 at the Wellcome Sanger Institute (WSI) Scientific Operations core from the whole organism using the Qiagen MagAttract HMW DNA kit, according to the manufacturer’s instructions. RNA from abdomen tissue of idEriArbu2 was extracted in the Tree of Life Laboratory at the WSI using TRIzol, according to the manufacturer’s instructions. RNA was then eluted in 50 μl RNAse-free water and its concentration assessed using a Nanodrop spectrophotometer and Qubit Fluorometer using the Qubit RNA Broad-Range (BR) Assay kit. Analysis of the integrity of the RNA was done using the Agilent RNA 6000 Pico Kit and Eukaryotic Total RNA assay.

### Sequencing

Pacific Biosciences HiFi circular consensus and 10X Genomics Chromium read cloud sequencing libraries were constructed according to the manufacturers’ instructions. Poly(A) RNA-Seq libraries were constructed using the NEB Ultra II RNA Library Prep kit. Sequencing was performed by the Scientific Operations core at the Wellcome Sanger Institute on Pacific Biosciences SEQUEL II (HiFi), Illumina HiSeq X (10X) and Illumina HiSeq 4000 (RNA-Seq) instruments. Hi-C data were generated from remaining head/thorax tissue of idEriArbu1 using the Arima v2 Hi-C kit in the Tree of Life laboratory and sequenced at the Scientific Operations core on an Illumina NovaSeq 6000 instrument.

### Genome assembly

Assembly was carried out with Hifiasm ( Cheng *et al.*, 2021); haplotypic duplication was identified and removed with purge_dups ( Guan *et al.*, 2020). One round of polishing was performed by aligning 10X Genomics read data to the assembly with longranger align, calling variants with freebayes ( Garrison & Marth 2012). The assembly was then scaffolded with Hi-C data ( Rao *et al.*, 2014) using SALSA2 ( Ghurye *et al.*, 2019). The assembly was checked for contamination as described previously ( Howe *et al.*, 2021). Manual curation was performed using HiGlass ( Kerpedjiev *et al.*, 2018) and Pretext. The mitochondrial genome was assembled using MitoHiFi ( Uliano-Silva *et al.*, 2021), which performs annotation using MitoFinder ( Allio *et al.*, 2020). The genome was analysed and BUSCO scores generated within the BlobToolKit environment ( Challis *et al.*, 2020). Table 3 contains a list of all software tool versions used, where appropriate.

**Table 3.** Software tools used.

Software tool	Version	Source
Hifiasm	0.15.1	Cheng *et al*., 2021
purge_dups	1.2.3	Guan *et al*., 2020
SALSA2	2.2	Ghurye *et al*., 2019
longranger align	2.2.2	https://support.10xgenomics.com/genome-exome/software/pipelines/latest/advanced/other-pipelines
freebayes	1.3.1-17-gaa2ace8	Garrison & Marth, 2012
MitoHiFi	2	Uliano-Silva *et al*., 2021
HiGlass	1.11.6	Kerpedjiev *et al*., 2018
PretextView	0.2.x	https://github.com/wtsi-hpag/PretextView
BlobToolKit	2.6.4	Challis *et al*., 2020

## Data availability

European Nucleotide Archive: Eristalis arbustorum (plane-faced dronefly). Accession number PRJEB46302; https://identifiers.org/ena.embl/PRJEB46302.

The genome sequence is released openly for reuse. The *E. arbustorum* genome sequencing initiative is part of the Darwin Tree of Life (DToL) project. All raw sequence data and the assembly have been deposited in INSDC databases. The genome will be annotated using the RNA-Seq data and presented through the Ensembl pipeline at the European Bioinformatics Institute. Raw data and assembly accession identifiers are reported in Table 1.

## Author information

Members of the University of Oxford and Wytham Woods Genome Acquisition Lab are listed here: https://doi.org/10.5281/zenodo.5746938.

Members of the Darwin Tree of Life Barcoding collective are listed here: https://doi.org/10.5281/zenodo.5744972.

Members of the Wellcome Sanger Institute Tree of Life programme are listed here: https://doi.org/10.5281/zenodo.5744840.

Members of Wellcome Sanger Institute Scientific Operations: DNA Pipelines collective are listed here: https://doi.org/10.5281/zenodo.5746904.

Members of the Tree of Life Core Informatics collective are listed here: https://doi.org/10.5281/zenodo.5743293.

Members of the Darwin Tree of Life Consortium are listed here: https://doi.org/10.5281/zenodo.5638618.
