# Supplementary material for: The genome sequence of the plain-faced dronefly, Eristalis arbustorum (Linnaeus, 1758)
Source: Wellcome Open Res. 2022 Feb 15;7:61. [Version 1] doi: 10.12688/wellcomeopenres.17580.1 (PMC11109569; doi:10.12688/wellcomeopenres.17580.1)
Supplement: Peer review history

# Reviewer report: Reviewer response for version 1

**DOI:** 10.21956/wellcomeopenres.19439.r70944

**Published:** 2024-05-13

**Authors:** Abby Davis

**Affiliations:** 1 The University of New England, NSW, Australia

This data report assembles the genome of the fly *Eristalis arbustorum, *a syrphid fly capable of providing dual ecosystem services to the environment. The authors describe how the fly representatives were acquired, how the DNA and RNA of the specimens were extracted, and how these data were sequenced. The genome data supplied here will be important to syrphid fly phylogenetics and taxonomic research.

 The rationale for creating the dataset is clearly described in the Background, the protocols for constructing this dataset are appropriate, the authors have listed sufficient details of methods and materials to allow replication by others, and the raw datasets have been deposited in the INSDC databases with the genome sequence of *E. arbustorum *free to use.

Are sufficient details of methods and materials provided to allow replication by others?

Yes

Is the rationale for creating the dataset(s) clearly described?

Yes

Are the datasets clearly presented in a useable and accessible format?

Yes

Are the protocols appropriate and is the work technically sound?

Yes

Reviewer Expertise:

Syrphidae, Diptera taxonomy

I confirm that I have read this submission and believe that I have an appropriate level of expertise to confirm that it is of an acceptable scientific standard.

# Reviewer report: Reviewer response for version 1

**DOI:** 10.21956/wellcomeopenres.19439.r48736

**Published:** 2022-04-25

**Authors:** Zhao Le

**Affiliations:** 1 School of Biological Sciences and Engineering, Shaanxi University of Technology, Hanzhong, China

The manuscript ‘The genome sequence of the plain-faced dronefly, *Eristalis arbustorum* (Linnaeus, 1758)’ applies the Hifi long read technology to assemble a species of syrphid flies, which have overwhelming importance for ecological stability and agricultural economy. To date, the available high-quality genome data of syrphids are limited, this newly generated data will contribute to the syrphid research.

 I have some comments:

- Although this is a paper aimed at the data report, the descriptions of results are too short for understanding. The authors presented several figures and tables in the paper, but almost none of them have been well explained and discussed in the current version manuscript. For readers, the meaning of each table and figures are quite confused. At least, the authors need to discuss some important parameters and results shown in each plot or table respectively to reflect the quality of this assembly version, or if there are some other available previous published genomes of closed species, it is worth doing a comparison.  

Overall, it feels like an unfinished manuscript and quite a lot of content needs to be filled.

Are sufficient details of methods and materials provided to allow replication by others?

Partly

Is the rationale for creating the dataset(s) clearly described?

Yes

Are the datasets clearly presented in a useable and accessible format?

Yes

Are the protocols appropriate and is the work technically sound?

Yes

Reviewer Expertise:

Syrphidae, genome, evolution

I confirm that I have read this submission and believe that I have an appropriate level of expertise to confirm that it is of an acceptable scientific standard.
